# From Early Stress to Adolescent Struggles: How Maternal Parenting Stress Shapes the Trajectories of Internalizing, Externalizing, and ADHD Symptoms

**DOI:** 10.3390/pediatric17040076

**Published:** 2025-07-18

**Authors:** Katerina Koutra, Chrysi Mouatsou, Katerina Margetaki, Georgios Mavroeides, Mariza Kampouri, Lida Chatzi

**Affiliations:** 1 Department of Psychology, School of Social Sciences, University of Crete, 74150 Rethymno, Crete, Greece; chrysamouatsou@gmail.com (C.M.); mavroeides@gmail.com (G.M.); 2 Clinic of Ρreventive and Social Medicine, Department of Social Medicine, Faculty of Medicine, University of Crete, 71003 Heraklion, Crete, Greece; katmargetaki@hotmail.com; 3 Institute of Environmental Medicine, Karolinska Institutet, 17177 Stockholm, Sweden; mariza.kampouri@ki.se; 4 Department of Preventive Medicine, Keck School of Medicine, University of Southern California, Los Angeles, CA 90033, USA; chatzi@usc.edu

**Keywords:** maternal parenting stress, internalizing symptoms, externalizing symptoms, attention deficit hyperactivity disorder, trajectories, Rhea cohort study

## Abstract

Background/Objectives: Parenting stress, the emotional strain resulting from the demands of child-rearing, can profoundly affect both parental well-being and children’s emotional and behavioral development. This study examined the impact of maternal parenting stress during early childhood on the longitudinal progression of children’s internalizing, externalizing, and attention deficit hyperactivity disorder (ADHD) symptoms, from early childhood through adolescence. Methods: The study included 406 mother–child pairs from the Rhea mother–child cohort in Crete, Greece. Maternal parenting stress was assessed at age 4 using the Parental Stress Scale (PSS). Children’s symptoms were evaluated at ages 4 (Strengths and Difficulties Questionnaire, ADHD Test), 6, 11, and 15 years (Child Behavior Checklist, Conners’ Parent Rating Scale) through maternal reports. Multivariate mixed regression models, incorporating a random intercept for each child and a random slope for age at follow-up, were used to analyze the trajectories of symptoms from ages 4 to 15. Group-based trajectory modeling was applied to identify trajectory groups from 4 to 15 years, and multinomial logistic regression models were implemented to examine the associations between parental stress and group trajectories. Results: The results revealed that higher parental stress at age 4 was significantly associated with increased internalizing (b = 0.94, 95% CI: 0.68, 1.21), externalizing (b = 1.03, 95% CI: 0.75, 1.30), and ADHD symptoms (b = 0.86, 95% CI: 0.58, 1.14) over the study period. Notably, the impact of parenting stress on behavioral problems decreased with age (interaction with age, *p* = 0.032). Additionally, higher parenting stress at age 4 was linked to a greater likelihood of belonging to adverse symptom trajectories, including high decreasing, low increasing, and stable high trajectories for both internalizing and externalizing problems. Conclusions: These findings underscore the importance of early maternal parenting stress as a predictor of long-term emotional and behavioral difficulties in children, emphasizing the need for early intervention programs that support maternal mental health and children’s emotional development.

## 1. Introduction

Children experiencing emotional and behavioral difficulties are at an increased risk for long-term adverse outcomes that can extend well into adulthood [1,2]. Internalizing and externalizing disorders are the most common mental health issues among children and adolescents, with prevalence rates of around 10% and 20%, respectively [3,4]. Internalizing symptoms, such as anxiety, depression, and social withdrawal, involve emotional distress directed inward, often leading to sadness, excessive worry, and difficulty engaging socially [5,6]. In contrast, externalizing symptoms, including defiance, inattention, hyperactivity, and aggression, are outwardly directed behaviors that disrupt social and academic functioning [5,7]. The terms “internalizing” and “externalizing” reflect a widely used, evidence-based framework for categorizing psychological disorders in youth. Internalizing disorders typically encompass anxiety and depressive disorders, while externalizing disorders include conditions such as conduct disorder, oppositional defiant disorder, and attention deficit hyperactivity disorder (ADHD). ADHD is the most prevalent and frequently diagnosed condition, marked by enduring patterns of inattention, hyperactivity, and impulsivity, which are associated with significant impairment and long-term challenges in education, employment, and social functioning [8]. The widespread nature of these concerns is evident in global epidemiological data, which estimate that 13% of children and adolescents worldwide experience mental health disorders [9]. In Greece, the prevalence is similarly concerning. A national study by Bibou-Nakou et al. [10] reported that 2–4% of Greek adolescents experience clinical emotional difficulties, 4–11% struggle with hyperactivity, and 5–10% face conduct problems.

Stressful environments have a profound impact on children’s emotional and behavioral well-being, especially during vulnerable developmental periods ranging from early childhood to adolescence. Parenting stress, defined as the distress experienced in response to challenging or frustrating interactions between parents and their children [11], is a common and significant challenge for many caregivers. This type of stress arises when parents feel overwhelmed by their caregiving responsibilities and can be categorized into two primary domains: the child domain, which involves stressors related to the child’s behaviors or characteristics, and the parent domain, which is linked to the parent’s emotional and psychological well-being [12]. Early childhood is a pivotal phase for emotional and behavioral development, marked by rapid brain growth and heightened sensitivity to environmental influences [13]. The development of neural circuits responsible for emotion regulation, including the prefrontal cortex and limbic structures, occurs predominantly during these early years [14]. Positive interactions with caregivers during this time foster secure attachments and adaptive emotional responses, while negative experiences can predispose children to emotional and behavioral challenges [15].

Research indicates that parenting stress can lower parental responsiveness and sensitivity—both essential for healthy child development—further aggravating the difficulties children face in these formative years [16]. Parenting stress has been consistently identified as a key factor influencing various child outcomes, including behavioral problems, attention difficulties, and aspects of cognitive development [17,18]. High levels of parental stress are linked to poor parenting practices, which in turn contribute to increased child adjustment difficulties, underscoring the interconnected nature of family stress and child mental health [19]. Specifically, elevated stress can impair a parent’s ability to implement effective strategies, resulting in reduced warmth, inconsistent discipline, and harsher parenting practices [20,21]. This dynamic is particularly problematic, as research shows that parental stress is associated with both externalizing behaviors, such as aggression and hyperactivity [22,23], and internalizing problems, like anxiety and depression [24,25]. A recent meta-analysis by van Dijk et al. [26] found a significant connection between parental stress and emotional and behavioral problems in school-age children, highlighting the importance of addressing parental stress to prevent long-term mental health issues in children.

Although much of the research on parenting stress has relied on cross-sectional designs, existing longitudinal studies have provided valuable insights into the long-term effects of parental stress on child behavior. For example, Kiff et al. [27] followed children over several years and found that persistent parenting stress was linked to ongoing externalizing and internalizing problems, highlighting the importance of early intervention to prevent the development of long-term behavioral issues. Similarly, Mackler et al. [28] examined the reciprocal relationships between parental stress and child externalizing behaviors from ages 4 to 10, confirming that these factors influence each other over time in a dynamic manner. Further supporting this idea, studies by Neece et al. [18] and Stone et al. [29] found that increased parenting stress led to greater externalizing behaviors in children, while higher levels of child externalizing behaviors also contributed to elevated parental stress later on. Extending these findings into adolescence, de Maat et al. [30] investigated both maternal and paternal parenting stress at multiple points during adolescence, showing that maternal stress at age 13 predicted behavioral problems in offspring at age 18, thus shedding light on the temporal nature of these effects. While longitudinal research on this topic remains limited, these studies collectively underscore the reciprocal and evolving relationship between parenting stress and child behavior, reinforcing the need for targeted interventions that address parenting stress early and continuously throughout a child’s development.

As previously documented, parenting stress has long been recognized as a significant contributor to child behavior problems, with numerous studies consistently highlighting its association with both internalizing and externalizing symptoms [22,25]. A systematic review and meta-analysis by Barroso et al. [31] further underscores that parenting stress is particularly pronounced among parents of children with developmental delays, autism spectrum disorder, and other clinical conditions. This review also found that parenting stress is more strongly linked to externalizing behaviors such as aggression and hyperactivity than to internalizing symptoms like anxiety and depression. However, despite this well-established connection in clinical populations, there is a notable gap in research regarding non-clinical groups. Addressing this gap, the present study aims to explore the impact of maternal parenting stress during early childhood on the long-term development of both internalizing and externalizing outcomes in children, with a particular focus on identifying critical points for intervention. This understanding could significantly enhance clinical practices, offering insights into how to design more targeted interventions that not only address children’s behavioral issues but also alleviate parental stress, ultimately improving outcomes for both children and parents. Furthermore, this study is the first to simultaneously examine the role of maternal parenting stress in the developmental trajectories of children’s internalizing, externalizing, and ADHD symptoms, while also exploring group-based symptom trajectories over time. By integrating both individual symptom trajectories and group-level patterns, this research offers a comprehensive view of how maternal parenting stress influences the evolution of emotional and behavioral difficulties in children and how these challenges may unfold in distinct subgroups across developmental stages. This innovative approach enables a more nuanced interpretation of the long-term effects of maternal stress, highlighting the potential for tailored interventions based on individual and group trajectories, which can better address the unique needs of at-risk children and their families.

The aim of the present study is to examine how maternal parenting stress during early childhood affects the development and course of children’s emotional and behavioral symptoms from childhood through adolescence. The study focuses on both symptom levels and developmental trajectories across internalizing-, externalizing-, and ADHD-related difficulties. Specifically, we test the following hypotheses: (a) Higher levels of maternal parenting stress at age 4 will be associated with increased internalizing, externalizing, and ADHD symptoms in children over time; and (b) Higher maternal parenting stress at age 4 will predict an increased likelihood of children following adverse developmental trajectories (e.g., persistent or escalating patterns) of internalizing, externalizing, and ADHD symptoms.

## 2. Materials and Methods

### 2.1. Participants

This study is part of the ongoing longitudinal Rhea Study, a mother–child cohort based in Heraklion, Crete, Greece. The cohort was initiated by recruiting pregnant women around the 12th week of gestation during their first major ultrasound, from both public and private clinics in Heraklion. Recruitment occurred over a 12-month period from February 2007 to February 2008. To be eligible for participation, women had to meet the following criteria: (i) reside in the Heraklion prefecture, (ii) be older than 16 years, and (iii) have a sufficient understanding of the Greek language.

At the start of the Rhea Study, 1610 pregnant women consented to join the cohort. However, due to various factors such as miscarriages, stillbirths, multiple pregnancies, relocation, and withdrawals, the number of singleton pregnancies followed to delivery decreased to 1363. Participants were contacted twice during pregnancy: first at recruitment (around 12 weeks of gestation), then again in the third trimester (around 32 weeks), with a final contact at birth admission (around 38 weeks). During these visits, biological samples (blood, urine, and cord blood) were collected, medical records reviewed, physical exams conducted, and questionnaires administered to gather data on socio-demographic characteristics, diet, lifestyle, and mental health. Postnatal follow-ups took place at multiple stages: infancy (9 and 18 months), early childhood (4.2 years), mid-childhood (6.5 years), and pre-adolescence (11 years). These visits involved additional biological sample collection, clinical child examinations, neurodevelopmental assessments, and questionnaires addressing various physical and mental health factors. The latest follow-up occurred at age 15, as part of the IntExt Trajectories project. A detailed description of the cohort, follow-up visits, and measurement protocols is available in the study by Chatzi et al. [32].

**Figure 1:**
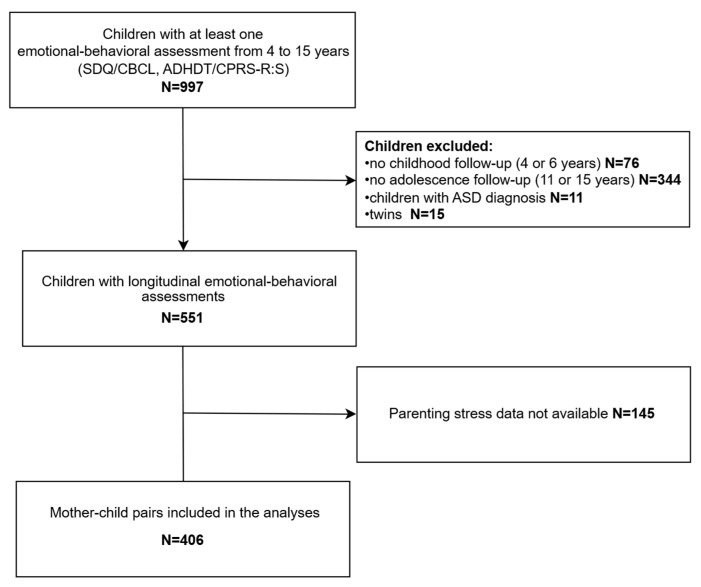
Flowchart of the study population. Abbreviations: ADHDT: Attention Deficit Hyperactivity Disorder Test; ASD: Autism Spectrum Disorder; CBCL: Child Behavior Checklist; CPRS-R:S: Conners’ Parent Rating Scale-Revised: Short Form; SDQ; Strengths and Difficulties Questionnaire.

The study examined emotional and behavioral development using longitudinal data collected at ages 4, 6, 11, and 15. Initially, 997 children with at least one assessment were included, but only those with data from both childhood (age 4 or 6) and adolescence (age 11 or 15) were retained. After excluding twins (*n* = 15) and children with autism spectrum disorder (*n* = 11), longitudinal emotional and behavioral assessments were available for 551 children. Of those, 145 children lacked data on maternal parenting stress at age 4. Thus, the final sample consisted of 406 mother–child pairs (Figure 1).

### 2.2. Measures

#### 2.2.1. Maternal Parenting Stress

To assess maternal stress related to parenting, the study utilized the Parental Stress Scale (PSS) [33] during the 4-year follow-up of the Rhea cohort. This 18-item self-report measure captures the complex emotional experience of parenting by balancing both its rewarding and challenging aspects. Items reflect positive dimensions, such as fulfillment and emotional enrichment, as well as negative dimensions, including stress, lack of control, and the strain on personal resources. Mothers responded on a 5-point Likert scale ranging from “strongly disagree” to “strongly agree”, with higher scores reflecting greater perceived stress. The PSS is a widely validated tool [33,34], and for the purposes of this study, it was carefully translated and culturally adapted by the research team to ensure its validity and applicability within the Greek population.

#### 2.2.2. Children’s Emotional, Behavioral and ADHD Difficulties

To assess children’s emotional-, behavioral-, and ADHD-related difficulties across development, a series of validated, age-appropriate parent-report instruments were administered as part of the Rhea cohort study. At the 4-year follow-up, mothers completed the Strengths and Difficulties Questionnaire (SDQ) [35], a 25-item tool that measures emotional symptoms, conduct problems, hyperactivity, peer problems, and prosocial behavior. For analysis, composite scores were calculated for internalizing difficulties (emotional symptoms + peer problems), and externalizing difficulties (conduct problems + hyperactivity), and Total difficulties. The SDQ was adapted for the Greek population [36]. For follow-up assessments at ages 6, 11, and 15, mothers completed the Child Behavior Checklist (CBCL) [37], a widely used 113-item measure assessing both adaptive and maladaptive behaviors. The CBCL summarizes children’s emotional and behavioral symptoms using two main approaches: empirically based syndrome scales and DSM-oriented scales. Key summary scores include Internalizing problems (anxiety/depression, withdrawal, somatic complaints), and Externalizing problems (rule-breaking, aggression), and Total problems. The Greek version of the CBCL [38] has demonstrated strong psychometric properties. For this analysis, only the internalizing and externalizing indices were used, with higher scores indicating more severe problems. Both instruments assess core aspects of internalizing problems, including sadness, fearfulness, depressive symptoms and excessive worry, as well as externalizing problems, including oppositional behavior and conduct issues.

In parallel, ADHD symptoms were evaluated using instruments aligned with DSM criteria. At age 4, mothers completed the Attention Deficit Hyperactivity Disorder Test (ADHDT) [39], a 36-item scale yielding subscale scores for Hyperactivity, Inattention, and Impulsivity, as well as a Total ADHD difficulties index. This tool was previously translated and validated in Greek [40]. At ages 6, 11, and 15, mothers completed the Conners’ Parent Rating Scale-Revised: Short Form (CPRS-R:S) [41], a 27-item scale assessing Oppositional behavior, Cognitive problems/Inattention, and Hyperactivity, along with a Total ADHD symptoms index. The CPRS-R:S was carefully translated and culturally adapted into Greek by the Rhea research team. For the present analyses, the total ADHD indices from both instruments were utilized, with higher scores indicating greater severity of ADHD symptoms. Indices from both instruments capture the primary behavioral characteristics of ADHD, namely inattention and hyperactivity.

### 2.3. Procedure

Parental stress was evaluated at the 4-year follow-up using maternal self-reports, while children’s emotional and behavioral development was evaluated longitudinally by trained psychologists through validated, parent-completed questionnaires. Prior to each assessment wave, mothers were contacted by phone, informed about the study’s aims, and invited to participate. Data collection involved face-to-face sessions during follow-up assessments at ages 4, 6, and 11 years. In the most recent follow-up at age 15 (IntExt Trajectories project), participants had the option to complete the assessments either face-to-face or via a secure digital platform. Full study details were provided, and written informed consent was obtained at each stage, initially from mothers and, during adolescence, also from the children themselves. Upon completing their participation, families received individualized feedback reports on the child’s psychological development, along with the option of a counseling session with the Rhea study’s psychological team. The study adhered to the ethical principles outlined in the Helsinki Declaration, with approvals granted by the Ethics Committee of the University Hospital of Heraklion (reference number: 96/06-02-2007) and the Research Ethics Committee of the University of Crete (reference number: 43/16-03-2022).

### 2.4. Statistical Analysis

Descriptive statistics were used to summarize sample characteristics, exposure variables, and study outcomes. Continuous variables were presented as means with standard deviations (SD), and categorical variables as frequencies with percentages. Normality of continuous variables was assessed using the Shapiro–Wilk test, and non-parametric methods were applied when appropriate. Bivariate associations between psychometric scale scores and participant characteristics were examined using Mann–Whitney U or Kruskal–Wallis tests for categorical variables, and Spearman’s correlation for continuous variables.

For missing data in outcomes, total scores were prorated when fewer than 25% of items were missing on a given scale. While different instruments were employed at the 4-year follow-up, the SDQ and CBCL both assess internalizing and externalizing symptoms, while the ADHDT and CPRS-R:S both capture the core features of ADHD (i.e., inattention and hyperactivity). As such, the instruments evaluate comparable constructs. To account for differences in scoring scales, we also applied statistical harmonization procedures. To harmonize outcome measures, percentiles were calculated for internalizing, externalizing, and ADHD symptom scales and used in all analyses.

To assess the association between maternal parenting stress and the longitudinal trajectories of emotional, behavioral, and ADHD symptoms from ages 4 to 15, multivariate mixed-effects regression models were employed. These models included a random intercept for each child and a random slope for age to account for within-child variability over time. To explore potential time-varying effects, interaction terms between maternal parenting stress and child age at follow-up were added. Effect modification by child sex was examined by including interaction terms between maternal parenting stress and child sex. Age-specific and sex-specific estimates were derived from these modes, as well as *p*-values for the interactions. Results are presented as beta coefficients and 95% confidence intervals (CI). Multivariable models included confounders identified through bivariate analysis (*p* < 0.20) as well as a priori covariates. Covariate collinearity was evaluated using the variance inflation factor (VIF < 10). Adjustments were made for child sex, exact age at assessment, maternal age at delivery, maternal smoking during pregnancy, gestational age or preterm birth, breastfeeding duration, parental education level, birth order, and area of residence (urban/rural) or maternal employment status during pregnancy. Sensitivity analyses were conducted by excluding children born preterm (<37 weeks of gestation) or with low birth weight (<2500 g). Additional analyses excluded children diagnosed with ADHD or a learning disability to assess robustness.

To identify distinct developmental patterns of internalizing, externalizing, and ADHD symptoms, Group-Based Trajectory Modeling (GBTM) was applied. All prorated scales were categorized into four groups representing degrees of severity. We defined the degrees of severity as follows: no symptoms (scores below the 50th percentile), low symptoms (scores between the 51st and 75th percentile), moderate symptoms (scores between the 76th and 90th percentile), and severe symptoms (scores above the 90th percentile) [42,43,44]. Trajectory models were estimated using the Stata Plugin traj, which computed (a) the probability of group membership, (b) the predicted trajectory for each group, and (c) the posterior probability of group membership. Symptom severity was modeled using a censored normal distribution, with censoring limits set beyond the observed data range. For each outcome, models specifying 2 to 4 trajectory groups were tested. The optimal model was selected based on several criteria: lowest Bayesian Information Criterion (BIC), group sizes greater than 5%, average posterior probabilities (APP) ≥ 0.70, odds of correct classification (OCC) > 5, and entropy values ≥ 0.80, indicating high classification certainty and good group separation [45,46].

Multivariate associations between maternal parenting stress and identified trajectory groups were examined using multinomial logistic regression, weighted by the individual’s posterior probability of group membership. Adjustments were made for child sex, exact age at assessment, maternal age at delivery, maternal smoking during pregnancy, gestational age or preterm birth, breastfeeding duration, parental education level, birth order, and area of residence (urban/rural) or maternal employment status during pregnancy. Separate models were constructed for each outcome, with the largest trajectory group (typically stable low symptoms) serving as the reference. Effect estimates are reported as relative risk ratios (RRRs) with 95% confidence intervals (CIs).

All hypothesis tests were two-sided with a significance level of 0.05. Analyses were conducted using Stata version 16.0 (StataCorp, College Station, TX, USA).

## 3. Results

### 3.1. Descriptives of the Study Population

The characteristics of the sample (N = 406) are presented in Table 1. On average, mothers were 30.1 years old (±4.6) and fathers were 34.1 years old (±5.6) at the time of childbirth. The majority of mothers had attained medium (50.4%) or high (39.5%) level of education, while the corresponding percentages for fathers were 43.7% and 27.5%. Most women were employed during pregnancy (79.7%), lived in urban areas (73.9%), and did not smoke during pregnancy (62.5%). Among children, 52.7% were male and 47.3% were female, 47 (11.6%) were born prematurely, and 44.2% were the firstborn in their families. Mean breastfeeding duration was 4.3 months (±4.1). By the age of 15 years, 21 children (5.2%) had been diagnosed with learning disabilities and 10 children (2.5%) had an ADHD diagnosis.

Participating mothers and fathers tended to have higher educational level. Mothers were also more likely to be employed during pregnancy and less likely to smoke during that period. In addition, participating families were more likely to be of Greek origin and reported higher household income. Children included in the analysis were less likely to be born prematurely, had slightly higher birth measurements, were breastfed for longer durations, and were more likely to have attended nursery before the age of 2 (Supplementary Table S1).

### 3.2. Descriptives of the Study Outcome Variables for the Total Sample and by Sex

The distribution of internalizing, externalizing, and ADHD symptoms is presented in Table 2. At age 15, females reported significantly more internalizing symptoms, while males had higher scores on externalizing problems at ages 4, 6, and 11 years. Males also consistently exhibited higher levels of ADHD symptoms than females at all timepoints.

### 3.3. Univariate Associations Between Maternal Parenting Stress and Internalizing, Externalizing and ADHD Symptoms at Ages 4, 6, 11, and 15 Years

Statistically significant positive correlations were found between maternal parenting stress and child outcomes at each assessment timepoint (Table 3).

### 3.4. Multivariate Associations Between Maternal Parenting Stress and Trajectories of Internalizing, Externalizing and ADHD Symptoms from 4 to 15 Years of Age, Mixed Model Analyses

Higher maternal parenting stress predicted increases in internalizing (b = 0.94, 95% CI: 0.68, 1.21), externalizing (b = 1.03, 95% CI: 0.75, 1.30), and ADHD-related symptoms (b = 0.86, 95% CI: 0.58, 1.14) over time (Table 4). Although higher maternal parenting stress was linked to greater behavioral difficulties, a significant interaction with age (*p* = 0.032) suggested that this association weakened as children grew older (Supplementary Table S2). No significant sex interaction was observed in the analyses, indicating that the associations between maternal parenting stress and child outcomes were similar across both male and female children (Supplementary Table S3).

Sensitivity analyses were also performed (Supplementary Table S4), excluding preterm infants, low birth weight children, and those diagnosed with learning disabilities or ADHD. These exclusions did not significantly alter the observed longitudinal associations between maternal parenting stress and child outcomes.

### 3.5. Multivariate Associations Between Maternal Parenting Stress and Trajectories of Internalizing, Externalizing and ADHD Symptoms from 4 to 15 Years of Age, Group-Based Trajectory Modeling

**Figure 2:**
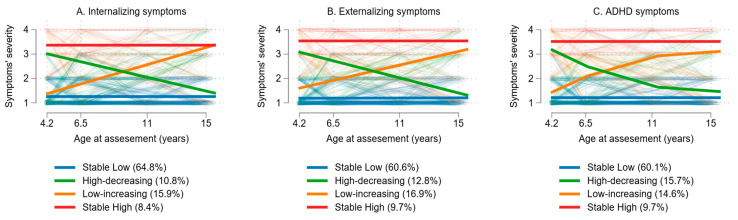
Internalizing (**A**), externalizing (**B**), and ADHD (**C**) symptom trajectories from early childhood through adolescence.

Using GBTM, we identified four distinct patterns of symptom development: stable low, high-decreasing, low-increasing, and stable high (Figure 2). The majority of children followed a stable low symptom trajectory (internalizing symptoms: 64.8%, externalizing symptoms: 60.6%, ADHD symptoms: 60.1%). A smaller proportion presented high symptoms in early childhood that declined over time (internalizing symptoms: 10.8%, externalizing symptoms: 12.8%, ADHD symptoms: 15.7%). Another group exhibited increasing symptoms from childhood to adolescence despite showing low symptoms during early childhood (internalizing symptoms: 15.9%, externalizing symptoms: 16.9%, ADHD symptoms: 14.6%). Finally, only a minority experienced persistently high symptoms over time (internalizing symptoms: 8.4%, externalizing symptoms: 9.7%, ADHD symptoms: 9.7%).

The associations between maternal parenting stress and trajectory groups are presented in Table 5. Increased maternal parenting stress at 4 years of child age was linked to elevated risk of being grouped in any of the adverse trajectories for internalizing symptoms (high decreasing: RRR [95% CI]: 1.04 [1.01, 1.07], low increasing: 1.04 [1.00, 1.07], stable high: 1.09 [1.04, 1.14]). Additionally, elevated parental stress, reported by mothers when children were 4 years old, was found to be associated with increased risk of membership in all adverse externalizing symptom trajectories (high decreasing: RRR [95% CI]: 1.07 [1.03, 1.11], low increasing: 1.05 [1.01, 1.08], stable high: 1.12 [1.07, 1.17]). Lastly, higher levels of parental stress at 4 years were associated with increased risk of children displaying high decreasing (RRR [95% CI]: 1.07 [1.03, 1.11]) and stable high ADHD symptoms (RRR [95% CI]: 1.10 [1.05, 1.15]). No sex interaction was found, indicating that the associations between maternal parenting stress and the likelihood of belonging to adverse symptom trajectories were consistent for both male and female children (Supplementary Table S5).

## 4. Discussion

The present study explored the long-term effects of maternal parenting stress on the developmental trajectories of internalizing, externalizing, and ADHD symptoms from early childhood to adolescence. To our knowledge, it is the first to examine the role of maternal parenting stress in early childhood and its influence on the progression of emotional and behavioral symptoms in offspring over time. Our findings indicate that maternal parenting stress was consistently associated with an increase in internalizing, externalizing, and ADHD symptoms in children. This suggests that maternal parenting stress in early childhood may be a key predictor of the development of a wide range of behavioral and emotional challenges. Interestingly, the effect of maternal parenting stress on behavioral problems diminished as children aged. The study also revealed that higher parenting stress at age 4 was associated with an increased likelihood of following negative symptom trajectories. This suggests that early interventions targeting maternal stress could play a crucial role in preventing or mitigating long-term emotional and behavioral difficulties in children.

Parenting stress refers to the emotional and psychological burden that arises when parents struggle to meet the demands of raising a child, often due to limitations in resources such as time, energy, or emotional support [47]. For mothers, this stress is particularly pronounced as they balance caregiving responsibilities, manage difficult child behaviors, and juggle various familial obligations. When support systems are scarce or children exhibit challenging behaviors, the strain can become overwhelming, potentially disrupting the parent–child relationship and influencing the child’s developmental trajectory. The first hypothesis of the study is confirmed as our findings underscore a strong, consistent association between maternal parenting stress in early childhood and an increase in internalizing, externalizing, and ADHD symptoms in children over time. These results align with previous longitudinal studies that have demonstrated the enduring impact of early environmental stressors on child development. More specifically, Mackler et al. [28] found that maternal parenting stress at age 4 was associated with externalizing symptoms in children across early and middle childhood, extending up to age 10. Similarly, a recent study by Chiang and Bai [48] demonstrated that maternal stress during early childhood (at age 5) was associated with the severity of internalizing symptoms at ages 9 and 15, as well as externalizing symptoms at age 9. Moreover, the identification of parenting stress as a key factor in child symptom trajectories suggests that intervention efforts targeting maternal stress may be a valuable strategy in preventing or mitigating the emergence of emotional and behavioral problems in at-risk children [14].

One interpretation of these findings is that high levels of maternal parenting stress create an emotionally charged and unstable home environment, which may contribute to the development or exacerbation of emotional and behavioral issues in children. The negative impact of this stress could manifest in various ways, such as difficulties with emotional regulation [49], heightened anxiety and depressive symptoms [50], or increased oppositional behaviors [51]. Additionally, maternal parenting stress may lead to changes in parenting behaviors, such as heightened anger and aggression [52,53], decreased responsiveness [54], and reduced emotional involvement [49], all of which could negatively influence a child’s emotional development. Less warmth, more psychological control, and inconsistent discipline [55] might further disrupt the child’s emotional regulation and behavior. Another plausible explanation is that children, especially in their early years, tend to model their behaviors after their primary caregivers. Cognitive behavioral models of psychopathology suggest that children internalize their mothers’ thoughts, emotions, and stress responses, which can shape the child’s own emotional and behavioral patterns [56]. If mothers display stress-related behaviors, such as frustration, avoidance, or emotional dysregulation, children may internalize these patterns as they develop their own coping mechanisms. According to the socio-cognitive theory, this process of modeling can lead to the emergence of similar emotional or behavioral difficulties in children as they adopt maladaptive coping strategies or become more susceptible to stress themselves. In addition, drawing on attachment theory and family systems perspective, it can be hypothesized that mothers who struggle with stress and feel overwhelmed by parenting demands may limit opportunities for emotional connection or hinder open communication with their children. This disruption in parent–child relationship may, in turn, increase the risk of emotional and behavioral difficulties in children.

Interestingly, the impact of maternal parenting stress on behavioral problems appeared to diminish as children aged. This age-related change in the trajectory of stress effects may be attributed to several factors. As children grow older, they gain increased cognitive and emotional capacities, allowing them to better regulate their emotions and behavior independently [57,58]. Additionally, external factors such as peer relationships, school environments, and broader social networks enhance resilience [59,60] and, thus, may buffer the direct impact of parental stress, offering children opportunities for positive social and emotional development. These external sources of support may gradually reduce the influence of maternal stress, allowing children to navigate stressors with greater competence as they mature. However, the diminishing effect of maternal stress on children’s behavioral problems with age should not be interpreted as a decline in the importance of maternal parenting stress over time. Instead, it highlights the dynamic nature of child development, where the interplay between early caregiving experiences and developmental milestones continually shapes children’s emotional and behavioral outcomes. While the direct influence of maternal parenting stress may lessen, its long-term effects can still be felt, albeit in more subtle or indirect ways. This reinforces the need for interventions that address maternal stress during early childhood but also emphasizes the importance of ongoing support as children grow, particularly for those who show signs of vulnerability due to early-life stressors. By providing continued support and resources, we can better equip children to manage emerging emotional and behavioral challenges throughout their development.

Finally, in line with the second hypothesis of the study, high maternal parenting stress at age four was identified as a significant risk factor for following adverse developmental trajectories of internalizing, externalizing, and ADHD symptoms, including high-decreasing, low-increasing, and stable high symptom patterns. While we were unable to identify previous studies specifically examining maternal parenting stress and offspring symptom trajectories using group-based analyses, a recent study by Kjeldsen et al. [61] found that various family-related stressors and low mother-reported support were associated with a stable high trajectory of offspring externalizing symptoms from early childhood to adolescence. In our study, the findings suggest that when mothers perceive the demands of parenting as overwhelming, it is linked to the development of persistent and escalating internalizing, externalizing, and ADHD symptomatology in their children, lasting from early childhood into adolescence. A possible explanation for this connection is that maternal parenting stress during the sensitive early childhood period may disrupt key parenting behaviors, including responsiveness [54] and effective parent–child communication [62], as well as parent dyadic coping strategies [63]. These disruptions could undermine the child’s emotional regulation and behavioral development, ultimately contributing to long-term behavioral and emotional difficulties. Previous research has highlighted that stressors experienced by parents can interfere with positive parenting practices and family dynamics, which are crucial for healthy child development [63,64,65]. This underscores the importance of early interventions to address maternal stress and support positive parenting strategies, particularly in the early years, to prevent the emergence of maladaptive symptom trajectories in children.

The clinical implications of the study findings highlight the importance of early identification and support for families experiencing high parenting stress. Interventions such as psychoeducational and evidence-based parenting programs can equip mothers and fathers with practical tools and strategies to manage everyday parenting challenges effectively, thereby increasing self-efficacy and improving parenting practices. In addition, stress management programs can help parents develop adaptive coping skills and encourage the utilization of social support networks. Finally, providing support for the parental couple to promote open communication and mutual understanding can enhance family cohesion, which may buffer the negative impact of parenting stress on child development.

A key strength of this study is its longitudinal design, which allowed for the assessment of symptom trajectories from early childhood through adolescence, providing valuable insights into developmental patterns over time. The application of advanced statistical methods, such as mixed model analysis and GBTM, enhanced the robustness of the findings by capturing both individual variability and subgroup patterns. Additionally, the study applied validated psychometric instruments and carefully accounted for a wide range of potential confounders, increasing the validity of the observed associations. The large population-based sample also improves the generalizability of the results within the context studied. Finally, the study draws on data from the well-established Rhea mother–child cohort in Crete, strengthening its relevance and applicability to similar Mediterranean and European populations.

However, several limitations should be considered. First, maternal parenting stress and child outcomes were assessed using self-report questionnaires, which may be subject to reporting bias or shared method variance. Second, although a broad set of covariates was included, residual confounding from unmeasured factors (e.g., paternal stress, family dynamics, or genetic predispositions) cannot be entirely ruled out. Third, the use of different psychometric instruments across developmental stages may have introduced measurement inconsistencies, despite efforts to harmonize scores across timepoints. We acknowledge that differences in item format and scoring methods between the tools used at the 4-year follow-up and those employed in subsequent assessments may have contributed to measurement error variance. Although harmonization procedures were applied, the results should be interpreted with caution. Fourth, despite efforts to handle missing data and conduct sensitivity analyses, attrition over the long follow-up period may have introduced bias, particularly if drop-out was related to the exposure or outcomes. Fifth, we recognize that the use of different modes of data collection (i.e., face-to-face and online) across assessment points may have introduced variability in the evaluation of children’s emotional and behavioral difficulties. Another important limitation of the study is the lack of paternal data on parenting stress and involvement, which we believe would have offered valuable insights, given the well-established role of paternal figure in child developmental outcomes. Future studies should explore the role of paternal parenting stress, as well as other factors, such as mother–father interaction and perceived social support, which could mediate the relationship between parenting stress and child outcomes. Finally, findings may not be directly generalizable to populations with different cultural or socioeconomic contexts outside of the study setting.

## 5. Conclusions

In conclusion, this study underscores the enduring impact of maternal parenting stress on the developmental trajectories of internalizing, externalizing, and ADHD symptoms from early childhood to adolescence. The findings emphasize the critical importance of early interventions aimed at reducing maternal stress and enhancing parenting practices during the formative early years. By supporting mothers in managing stress and cultivating effective parenting strategies, these interventions could significantly reduce the risk of emotional and behavioral issues in children. Early intervention offers a valuable opportunity to disrupt the cycle of stress and maladaptive behaviors, fostering healthier parent–child relationships and promoting better long-term emotional and behavioral outcomes for children. Further research could investigate the most effective interventions for reducing maternal stress and the mechanisms by which these interventions influence child outcomes.

## Figures and Tables

**Table 1:** Parental and offspring characteristics of the sample (N = 406).

	N	% or Mean (SD)		N	% or Mean (SD)
**Maternal age at childbirth** (years)	404	30.1 (4.6)	**Child sex**		
**Paternal age at childbirth** (years)	400	34.1 (5.6)	Male	214	52.7
**Maternal education**			Female	192	47.3
Low	41	10.2	**Gestational age** (weeks)	404	38.2 (1.5)
Medium	203	50.4	**Preterm birth** (<37 weeks)		
High	159	39.5	Yes	47	11.6
**Maternal working status**			No	357	88.4
Employed	314	79.7	**Mode of delivery**		
Not working/Unemployed	80	20.3	Vaginal	207	51.0
**Maternal marital status**			Cesarian section	199	49.0
Married	362	90.7	**Birth anthropometry**		
Other	37	9.3	Weight (kg)	405	3.2 (0.4)
**Paternal education**			Length (cm)	405	50.6 (2.1)
Low	114	28.8	Head circumference (cm)	405	34.2 (1.3)
Medium	173	43.7	**Birth order**		
High	109	27.5	First	159	44.2
**Paternal working status**			Second	132	36.7
Employed	398	99.5	Third or more	69	19.2
Not working/Unemployed	2	0.5	**Breastfeeding duration** (months)	397	4.3 (4.1)
**Area of living**			**Nursery before 2 years**		
Urban	300	73.9	Yes	99	24.4
Rural	106	26.1	No	307	75.6
**Family origin**			**Exact age at assessment**		
Greek	382	95.3	4 years	406	4.2 (0.2)
Foreign/Mixed	19	4.7	6 years	323	6.5 (0.3)
**Household income** (tertiles)			11 years	246	10.9 (0.3)
Low (<830 €/month)	85	24.3	15 years	354	14.9 (0.4)
Middle (831–1157 €/month)	123	35.1	**Diagnosis**		
High (1158–2241 €/month)	142	40.6	None	375	92.4
**Parity**			Learning disabilities	21	5.2
Nulliparous	176	44.8	ADHD	10	2.5
Multiparous	217	55.2			
**Maternal smoking status during pregnancy**					
Never	248	62.5			
Ever	149	37.5			

**Table 2:** Distribution of the study outcomes (raw scores) for the total sample (N = 406) and by gender.

	Overall				Males		Females		
	N	Mean (SD)	Min	Max	N	Mean (SD)	N	Mean (SD)	*p*-Value
**Internalizing symptoms**									
SDQ 4 years	406	3.2 (2.3)	0	11	214	3.4 (2.4)	192	3.0 (2.2)	0.056
CBCL 6 years	320	5.9 (4.2)	0	19	172	6.2 (4.3)	148	5.5 (4.0)	0.098
CBCL 11 years	244	7.0 (5.4)	0	31	134	7.2 (5.6)	110	6.7 (5.3)	0.500
CBCL 15 years	352	6.7 (5.6)	0	29	181	5.9 (5.0)	171	7.6 (6.1)	**0.004**
**Externalizing symptoms**									
SDQ 4 years	405	5.4 (3.1)	0	16	214	5.8 (3.2)	191	4.9 (2.8)	**0.001**
CBCL 6 years	322	8.2 (6.3)	0	38	173	9.4 (6.6)	149	6.9 (5.6)	**<0.001**
CBCL 11 years	244	7.1 (6.5)	0	36	134	8.1 (7.5)	110	5.9 (4.9)	**0.010**
CBCL 15 years	351	6.3 (6.2)	0	42	181	6.5 (6.2)	170	6.1 (6.2)	0.539
**ADHD symptoms**									
ADHDT 4 years	405	14.9 (12.0)	0	62	214	16.6 (13.0)	191	13.0 (10.5)	**0.002**
CPRS 6 years	317	8.6 (5.3)	0	27	172	9.3 (5.4)	145	7.8 (5.1)	**0.008**
CPRS 11 years	246	8.2 (5.3)	0	28	135	8.9 (5.6)	111	7.3 (4.7)	**0.019**
CPRS 15 years	353	7.8 (5.8)	0	29	181	8.7 (5.8)	172	6.8 (5.5)	**0.002**

Abbreviations: ADHDT: Attention Deficit Hyperactivity Disorder Test; CBCL: Child Behavior Checklist; CPRS: Conners’ Parent Rating Scale; SDQ: Strengths and Difficulties Questionnaire. Bold font indicates *p* < 0.05.

**Table 3:** Correlations between maternal parenting stress and child internalizing, externalizing and ADHD symptom percentiles at ages 4, 6, 11 and 15 years.

	Maternal Parenting Stress at 4 Years		
	N	Rho	*p*-Value
**Internalizing symptoms**			
4 years	406	0.288	**<0.001**
6 years	320	0.229	**<0.001**
11 years	244	0.177	**0.006**
15 years	352	0.240	**<0.001**
**Externalizing symptoms**			
4 years	405	0.319	**<0.001**
6 years	322	0.318	**<0.001**
11 years	244	0.171	**0.008**
15 years	351	0.210	**<0.001**
**ADHD symptoms**			
4 years	405	0.271	**<0.001**
6 years	317	0.257	**<0.001**
11 years	246	0.151	**0.018**
15 years	353	0.215	**<0.001**

Bold font indicates *p* < 0.05.

**Table 4:** Adjusted associations of maternal parenting stress and internalizing, externalizing and ADHD symptoms across 4 to 15 years of age, mixed model analyses.

	Maternal Parenting Stress at 4 Years		
	Ν	b (95% CI)	*p*-Value
**Internalizing symptoms ^a^**			
4–15 years	380	0.94 (0.68, 1.21)	**<0.001**
**Externalizing symptoms ^b^**			
4–15 years	378	1.03 (0.75, 1.30)	**<0.001**
**ADHD symptoms ^b^**			
4–15 years	381	0.86 (0.58, 1.14)	**<0.001**

^a^ Adjusted for child sex and exact age at assessment, maternal age, maternal smoking during pregnancy, preterm birth, breastfeeding duration, maternal education, paternal education, birth order and urban area of living. ^b^ Adjusted for child sex and exact age at assessment, maternal age, maternal smoking during pregnancy, gestational age, breastfeeding duration, maternal education, paternal education, birth order and maternal working status. Bold font indicates *p* < 0.05.

**Table 5:** Adjusted associations of maternal parenting stress and trajectory groups of internalizing, externalizing and ADHD symptoms across ages 4 to 15 years, multivariate analyses.

		High Decreasing		Low Increasing		Stable High	
	N	RRR (95% CI)	*p*-Value	RRR (95% CI)	*p*-Value	RRR (95% CI)	*p*-Value
**Internalizing symptoms ^a^**							
Parenting stress 4 years	380	1.04 (1.01, 1.07)	**0.012**	1.04 (1.00, 1.07)	**0.036**	1.09 (1.04, 1.14)	**<0.001**
**Externalizing symptoms ^b^**							
Parenting stress 4 years	378	1.07 (1.03, 1.11)	**<0.001**	1.05 (1.01, 1.08)	**0.008**	1.12 (1.07, 1.17)	**<0.001**
**ADHD symptoms ^b^**							
Parenting stress 4 years	381	1.07 (1.03, 1.11)	**<0.001**	1.02 (0.99, 1.06)	0.209	1.10 (1.05, 1.15)	**<0.001**

^a^ Adjusted for child sex and exact age at assessment, maternal age, maternal smoking during pregnancy, preterm birth, breastfeeding duration, maternal education, paternal education, birth order and urban area of living. ^b^ Adjusted for child sex and exact age at assessment, maternal age, maternal smoking during pregnancy, gestational age, breastfeeding duration, maternal education, paternal education, birth order and maternal working status. Notes: Reference group: Stable Low Trajectory. Bold font indicates *p* < 0.05.

## Data Availability

Data supporting these findings are available upon request from the corresponding author K.K.

## Supplementary Materials

The following supporting information can be downloaded at: https://www.mdpi.com/article/10.3390/pediatric17040076/s1, Table S1: Non-response analysis; Table S2: Age interaction and adjusted associations of maternal parenting stress and internalizing, externalizing and ADHD symptoms across 4 to 15 years of age, mixed model analyses; Table S3: Sex interaction and adjusted associations of maternal parenting stress and internalizing, externalizing and ADHD symptoms across 4 to 15 years of age, mixed model analyses; Table S4: Sensitivity analyses, mixed models; Table S5: Sex interaction and adjusted associations of maternal parenting stress and trajectory groups of internalizing, externalizing and ADHD symptoms across ages 4 to 15 years, multivariate analyses.

## Abbreviations

The following abbreviations are used in this manuscript:

ADHD	Attention Deficit Hyperactivity Disorder
ADHDT	Attention Deficit Hyperactivity Disorder Test
APP	Average Posterior Probabilities
BIC	Bayesian Information Criterion
CBCL	Child Behavior Checklist
CIs	Confidence intervals
GBTM	Group-Based Trajectory Modeling
CPRS-R:S	Conners’ Parent Rating Scale-Revised: Short Form
OCC	Odds of Correct Classification
PSS	Parental Stress Scale
RRRs	relative risk ratios
SDQ	Strengths and Difficulties Questionnaire
