# Supplementary material for: From Early Stress to Adolescent Struggles: How Maternal Parenting Stress Shapes the Trajectories of Internalizing, Externalizing, and ADHD Symptoms
Source: Pediatr Rep. 2025 Jul 18;17(4):76. doi: 10.3390/pediatric17040076 (PMC12286040; doi:10.3390/pediatric17040076)
Supplement: Supplementary file 1 [file pediatrrep-17-00076-s001.zip › pediatrrep-3648097-supplementary.pdf]

Supplementary Table S1. Non-response analysis.

	Participants N=406		Non-participants N=591		
	**N**	**% or Mean (SD)**	**N**	**% or Mean (SD)**	**p-value**
**Maternal age at childbirth** (years)	404	30.1 (4.6)	585	29.8 (5.2)	0.328
**Paternal age at childbirth** (years)	400	34.1 (5.6)	532	33.8 (5.5)	0.465
**Maternal education**					
Low	41	10.2	110	19.7	<0.001
Medium	203	50.4	292	52.3	
High	159	39.5	156	28.0	
**Maternal working status**					0.020
Employed	314	79.7	391	73.1	
Not working/ Unemployed	80	20.3	144	26.9	
**Maternal marital status**					0.089
Married	362	90.7	476	87.2	
Other	37	9.3	70	12.8	
**Paternal education**					0.032
Low	114	28.8	196	36.4	
Medium	173	43.7	222	41.3	
High	109	27.5	120	22.3	
**Paternal working status**					0.101
Employed	398	99.5	532	98.3	
Not working/ Unemployed	2	0.5	9	1.7	
**Area of living**					0.206
Urban	300	73.9	415	70.2	
Rural	106	26.1	176	29.8	
**Family origin**					0.041
Greek	382	95.3	500	91.9	
Foreign / Mixed	19	4.7	44	8.1	
**Household income** (tertiles)					<0.001
Low (<830 €/month)	85	24.3	173	40.8	
Middle (831-1157 €/month)	123	35.1	135	31.8	
High (1158-2241 €/month)	142	40.6	116	27.4	
**Parity**					0.987
Nulliparous	176	44.8	242	44.7	
Multiparous	217	55.2	299	55.3	
**Maternal smoking status during pregnancy**					0.017
Never	248	62.5	297	54.7	
Ever	149	37.5	246	45.3	
**Child sex**					0.937
Male	214	52.7	310	52.5	
Female	192	47.3	281	47.5	
**Gestational age** (weeks)	404	38.2 (1.5)	559	38.0 (1.8)	0.038
**Preterm birth** (<37 weeks)					0.021
Yes	47	11.6	95	17.0	
No	357	88.4	464	83.0	
**Mode of delivery**					0.126

Vaginal	207	51.0	260	46.0	
Cesarian section	199	49.0	305	54.0	
**Birth anthropometry**					
Weight (kg)	405	3.2 (0.4)	551	3.1 (0.5)	<0.001
Length (cm)	405	50.6 (2.1)	572	50.0 (2.8)	<0.001
Head circumference (cm)	405	34.2 (1.3)	572	33.9 (1.8)	0.006
**Birth order**					0.839
First	159	44.2	208	44.6	
Second	132	36.7	176	37.8	
Third or more	69	19.2	82	17.6	
**Breastfeeding duration** (months)	397	4.3 (4.1)	536	3.5 (4.2)	0.009
**Nursery before 2 years**					0.008
Yes	99	24.4	101	17.5	
No	307	75.6	477	82.5	

Supplementary Table S2. Age interaction and adjusted associations of maternal parenting stress and internalizing, externalizing and ADHD symptoms across 4 to 15 years of age, mixed model analyses.

		**4 years**		**6 years**		**11 years**		**15 years**		**p interaction**
	**N**	**b (95% CI)**	**p-value**	**b (95% CI)**	**p-value**	**b (95% CI)**	**p-value**	**b (95% CI)**	**p-value**	**with age**
**Internalizing symptoms** [a]										
Parental stress 4 years	380	1.07 (0.73, 1.42)	**<0.001**	0.96 (0.55, 1.36)	**<0.001**	0.68 (0.26, 1.10)	**0.002**	0.90 (0.52, 1.28)	**<0.001**	0.454
**Externalizing symptoms** [b]										
Parental stress 4 years	378	1.18 (0.86, 1.50)	**<0.001**	1.13 (0.73, 1.53)	**<0.001**	0.61 (0.18, 1.04)	**0.006**	0.75 (0.33, 1.16)	**<0.001**	**0.032**
**ADHD symptoms** [b]										
Parental stress 4 years	381	1.01 (0.65, 1.36)	**<0.001**	0.93 (0.55, 1.31)	**<0.001**	0.49 (0.04, 0.93)	**0.032**	0.77 (0.40, 1.15)	**<0.001**	0.154

[a] Adjusted for child sex and exact age at assessment, maternal age, maternal smoking during pregnancy, preterm birth, breastfeeding duration, maternal education, paternal education, birth order and urban area of living.

[b] Adjusted for child sex and exact age at assessment, maternal age, maternal smoking during pregnancy, gestational age, breastfeeding duration, maternal education, paternal education, birth order and maternal working status.

Bold font indicates $p < 0.05$.

Supplementary Table S3. Sex interaction and adjusted associations of maternal parenting stress and internalizing, externalizing and ADHD symptoms across 4 to 15 years of age, mixed model analyses.

		Males		Females		p interaction
	N	b (95% CI)	p-value	b (95% CI)	p-value	with sex
**Internalizing symptoms** [a]						
Parental stress 4 years	380	0.93 (0.58, 1.28)	**<0.001**	0.95 (0.55, 1.35)	**<0.001**	0.956
**Externalizing symptoms** [b]						
Parental stress 4 years	378	1.10 (0.74, 1.46)	**<0.001**	0.94 (0.52, 1.35)	**<0.001**	0.554
**ADHD symptoms** [b]						
Parental stress 4 years	381	0.87 (0.52, 1.22)	**<0.001**	0.85 (0.41, 1.28)	**<0.001**	0.930

[a] Adjusted for child sex and exact age at assessment, maternal age, maternal smoking during pregnancy, preterm birth, breastfeeding duration, maternal education, paternal education, birth order and urban area of living.

[b] Adjusted for child sex and exact age at assessment, maternal age, maternal smoking during pregnancy, gestational age, breastfeeding duration, maternal education, paternal education, birth order and maternal working status.

Bold font indicates $p < 0.05$.

Supplementary Table S4. Sensitivity analyses, mixed models.

	Excluding children born preterm		Excluding children with low birthweight		Excluding children with diagnosis of learning disability		Excluding children with diagnosis of ADHD	
	**N**	**b (95% CI)**	**N**	**b (95% CI)**	**N**	**b (95% CI)**	**N**	**b (95% CI)**
**Internalizing symptoms** [a]								
Parental stress 4 years	336	**0.94 (0.67, 1.22)**	363	**0.93 (0.65, 1.20)**	360	**0.95 (0.68, 1.22)**	370	**0.92 (0.65, 1.19)**
**Externalizing symptoms** [b]								
Parental stress 4 years	334	**1.04 (0.76, 1.33)**	361	**1.04 (0.76, 1.32)**	358	**1.05 (0.76, 1.34)**	368	**1.03 (0.76, 1.31)**
**ADHD symptoms** [b]								
Parental stress 4 years	336	**0.86 (0.57, 1.15)**	364	**0.87 (0.58, 1.15)**	361	**0.91 (0.63, 1.19)**	371	**0.84 (0.56, 1.11)**

[a] Adjusted for child sex and exact age at assessment, maternal age, maternal smoking during pregnancy, preterm birth, breastfeeding duration, maternal education, paternal education, birth order and urban area of living.

[b] Adjusted for child sex and exact age at assessment, maternal age, maternal smoking during pregnancy, gestational age, breastfeeding duration, maternal education, paternal education, birth order and maternal working status.

Bold font indicates $p < 0.05$.

Supplementary Table S5. Sex interaction and adjusted associations of maternal parenting stress and trajectory groups of internalizing, externalizing and ADHD symptoms across ages 4 to 15 years, multivariate analyses.

	N	Males			Females			p interaction with sex		
		High Decreasing	Low Increasing	Stable High	High Decreasing	Low Increasing	Stable High	High Decreasing	Low Increasing	Stable High
		RRR (95% CI)	RRR (95% CI)	RRR (95% CI)	RRR (95% CI)	RRR (95% CI)	RRR (95% CI)			
**Internalizing symptoms [a]**										
Parental stress 4 years	380	1.04 (1.00, 1.08)	1.02 (0.98, 1.07)	**1.09** (1.04, 1.16)**	1.04 (1.00, 1.08)	1.04 (1.00, 1.09)	**1.08* (1.00, 1.16)**	0.937	0.544	0.775
**Externalizing symptoms [b]**										
Parental stress 4 years	378	**1.08** (1.03, 1.13)**	**1.07** (1.02, 1.11)**	**1.12*** (1.06, 1.17)**	**1.05* (1.00, 1.11)**	1.02 (0.97, 1.07)	**1.14* (1.03, 1.27)**	0.530	0.164	0.719
**ADHD symptoms [b]**										
Parental stress 4 years	381	**1.05** (1.01, 1.10)**	1.00 (0.97, 1.04)	**1.07* (1.02, 1.13)**	**1.09** (1.03, 1.16)**	1.05 (0.98, 1.12)	**1.16*** (1.08, 1.24)**	0.309	0.245	0.090

[a] Adjusted for child sex and exact age at assessment, maternal age, maternal smoking during pregnancy, preterm birth, breastfeeding duration, maternal education, paternal education, birth order and urban area of living.

[b] Adjusted for child sex and exact age at assessment, maternal age, maternal smoking during pregnancy, gestational age, breastfeeding duration, maternal education, paternal education, birth order and maternal working status.

Notes: Reference group: Stable Low Trajectory.

* p < 0.05. ** p < 0.01. *** p < 0.001.

Bold font indicates p < 0.05.
